# Current State and Future Directions of Technologies for Music Instrument Pedagogy

**DOI:** 10.3389/fpsyg.2022.835609

**Published:** 2022-03-22

**Authors:** Alberto Acquilino, Gary Scavone

**Affiliations:** Computational Acoustic Modeling Laboratory, Center for Interdisciplinary Research in Music Media and Technology, Schulich School of Music, McGill University, Montreal, QC, Canada

**Keywords:** technology, learning, musicians, performance, edTech

## Abstract

Technological advances over the past 50 years or so have resulted in the development of a succession of hardware and software systems intended to improve the quality and effectiveness of Western music instrument pedagogy during classroom instruction or individual study. These systems have aimed to provide evaluation or visualization of single or combined technical aspects by analyzing performance data collected in real time or offline. The number of such educational technologies shows an ever-increasing trend over time, aided by the wide diffusion and availability of mobile devices. However, we believe there are unrealized opportunities for modern technologies to help music students in their technical development and assist them during their practice sessions in between visits to their teachers. The ubiquity of PCs and mobile devices with built-in microphones, speakers, and cameras has inspired the development of media technologies in support of music pedagogy. They offer an attractive potential for implementing audio signal processing algorithms addressing different technical skills of the performer, providing real-time feedback, collecting data over time, and applying statistical models. Despite this potential, most available software for music instrument pedagogy remains very limited in functionality. This study provides a survey of music edTech software available, together with the methods of use, addressed technical skills, commonalities, and limitations. Results show that most current software is based on the metronome and tuner, with only a few systems that have limited abilities to follow a performance in real-time and compare it to a given score to monitor correctness of notes, intonation, and rhythm. The survey also highlights a high and under-exploited potential regarding the monitoring of other more specific technical skills, which are more instrument-dependent, but no less important, such as the control of dynamic range and clarity of the attack. This article ends with a discussion of possible directions for future development of technologies to support the practice of music students at different levels, with some consideration for the corresponding signal processing methods that can be utilized or that need advancement. By helping students to more efficiently achieve a high level of proficiency of their instruments with assistive technologies, we hope to minimize stress and afford better enjoyment of the music performance experience for all.

## 1. Introduction

Musical pedagogy for learning traditional Western musical instruments is currently most often delivered in one-on-one or group contexts through a master-apprentice model, typically one time per week for 30–60 min per session (Hanken, 2017). Between those meetings, students practice on their own the assigned exercises and, attempting to apply the suggestions received in class, try to reach the learning goals set out by the instructor. It is a common problem that students either misunderstand or do not correctly remember the details of a performance technique (Welch, 1985), which can lead to frustration, slower development, and potentially termination of music studies.

Evidence from a wide variety of motor control tasks shows that real-time visual feedback can accelerate the learning progress (Shea and Wulf, 1999) and can help learners to identify, become aware of, or modify specific bodily actions (Welch et al., 2005). These findings suggest the development of technological tools based on audiovisual feedback to help music students address the aforementioned problems. Indeed, improvements in the effectiveness of learning classical music through aural and visual feedback has been demonstrated in different study applications (Ferguson, 2006; Leong and Cheng, 2014; Malandrino et al., 2019; Pardue and McPherson, 2019).

Among the oldest assistive technologies available for musical practice is the tuning fork, invented in 1711 by John Shore in London (Feldmann, 1997). Presenting a resonance frequency almost constant under any weather condition, this tool was used as a reference for tuning musical instruments. About a century later, the metronome was devised, providing a periodic “tick” sound at a desired tempo, typically in beats per minute, that can be set by the user. Patented for musical purposes in 1815 by Johann Maelzel, the metronome was proposed as a tool for composers, to indicate in a simple and objective way the speed of execution of their scores, and for music students, to develop a proper observance of time (Parker, 1825). More recently, based on the tuning fork principle, electronic tuners have become widely available and inexpensive, providing feedback on a player's intonation with respect to a particular tuning system, though they are often only used at the beginning of a practice session to make sure an instrument is correctly tuned.

The rapid spread of digital technologies with ever greater computational capabilities has made possible the continuous development of increasingly refined musical educational software. The metronome and tuner have been transformed from dedicated hardware devices to software that use the integrated components of PCs and mobile technologies. Furthermore, new functionalities and methods of interaction have been added that create greater engagement between the musician and the system.

A comprehensive survey on software for musicians and music teachers was provided by Axford (2015), although this field is constantly evolving and characterized by a high launch and dropout rate, making the list partially outdated after a few years. Despite there being a large number of software developed for music pedagogy in recent years, these systems appear to be underused due to interface inefficiency, technological complexity, and lack of institutional support (Kenny and McDaniel, 2011; Fautley, 2013; Gall, 2013). One might expect this situation to stem from a reluctant and conservative philosophy of thinking toward technology in music education (Creech and Gaunt, 2012; Gaunt, 2017). However, Waddell and Williamon (2019) found evidence of a generally positive attitude toward current and future technology use among teachers, amateur, students, and professional musicians. This also points to a general problem in perceived or actual effectivity of current software technologies for music pedagogy.

Musicians appear to be interested in integrating new technological tools into their practice routines and the ubiquity of mobile devices offers a convenient platform through which such tools can be made available. In this context, the present study provides a survey of existing technologies in the field of music education. By analyzing how they are structured, classifying them and discussing their pedagogical potential, we attempt to show their strengths and weaknesses, with the objective of providing an explanation regarding the gap between the wide availability of edTech music software and its relative under use in music education. We then discuss promising directions for future technologies in this field.

Section 2 outlines how educational technologies have been researched to assist music students. Section 3 presents a collection of the most common and innovative technologies in support of music education, proposing different classifications. Finally, in section 4 we discuss their pedagogical potential in music classrooms, highlighting strengths and weaknesses, in order to illustrate future directions in the development of educational technologies in this field.

## 2. Review of Musical Instrument Educational Technologies

The present study is focused on technologies that support music students in their development in learning to play a musical instrument. Such technologies are more applicable to the learning of standard technical skills (e.g., control of dynamics, articulation, intonation) rather than musical expression, which can be more subjective. Thus, it is expected that these tools will be more beneficial for beginning, rather than advanced, students as they work to develop basic functional skills on a given instrument. From a technological standpoint, we believe that tools designed to evaluate music learning from sound signals, rather than video or special purpose sensors, hold the most promise for widespread acceptance.

There have been several past academic research projects aimed at developing tools to assist with music instrument learning. A project to support piano instruction for beginner students was pursued during the 1980s and early 1990s, with reported achievements in polyphonic score following, page turning, analysis, feedback, and the application of Instructional Design theory (Dannenberg et al., 1993). Another study examined the effect on improving harmonic intonation skills, specifically the ability to play justly tuned major thirds on a reference tone, using Coda Music Technology's Intonation Trainer software program (Swift, 2003). This technology is based on the concept that musical instruments with variable pitch (e.g., strings, woodwinds, brass) can adjust their pitch as they are played. Players of these instruments are therefore released from the equal tempered intonation system and it becomes important for them to develop the ability to play chords with improved harmonic ratios (compared to equal-tempered tuning), and thus reduce beating effects. However, the idea did not find widespread adoption at a time when accessibility to a computer workstation and recording equipment was still limited to music students.

The Interactive Music Tuition System (IMUTUS) was a European project that ran from 2002 to 2005 with the goal of developing an open platform for training beginner students on the recorder (Tambouratzis et al., 2002; Raptis et al., 2005; Schoonderwaldt et al., 2005). It focused on score matching pitch and note onsets, with a user interface that “graded” students on their overall performance, indicated locations in the score where mistakes were made and provided some basic description on each error.

Another project was focused on the evaluation of saxophone performance using a system to track the fundamental playing frequency and perceived loudness level for specifically prescribed exercises consisting of long tones of both fixed and varying dynamic level (Robine and Lagrange, 2006; Percival et al., 2007; Robine et al., 2007; Percival, 2008). The use of such exercises helped avoid problems in distinguishing between technical errors and deliberate expressive decisions by performers, whereby they may intentionally nuance their playing to achieve expressive effects. The results of the analysis were reported to users *via* a simple computer interface, with additional features to allow comparison of results between other students in a class.

In the field of music information retrieval, a research project investigated the possibility of using machine learning algorithms to differentiate between good and poor quality trumpet notes (Knight et al., 2011). Each of the notes were analyzed and rated individually in a monophonic and unaccompanied context. Although the results of this study were not conclusive, the widespread application of artificial intelligence methods in nearly all computing contexts offers opportunities for the development of tools to provide useful feedback to students learning to play music instruments.

A more recent European Commission project, Technology Enhanced Learning of Music Instruments (TELMI, 2016–2019) included the design and implementation of new interaction paradigms for music learning and training based on state-of-the-art technologies (Kholykhalova et al., 2017; Ortega et al., 2017; Giraldo et al., 2019; Perez-Carrillo, 2019). The project focused primarily on violin performance, with the development of a prototype tool called SkyNote that can provide real-time feedback on pitch and intonation, dynamics, tone quality, and rhythm. When combined with a motion-tracking system, SkyNote can also monitor specific aspects of bowing technique including bow tilt, speed, weight, contact point, inclination, and direction. A recent project reported the use of an interactive robot for recorder tutoring (Bagga et al., 2019).

A limited number of technologies have been commercially developed to assist with general music learning, such as software systems for music theory, ear and rhythm training, music notation, and music instrument practice. In section 3, we provide an overarching overview of these software, analyzing classifications between them and examining their functions. In section 4, the potential limitations of such software and the possible future directions from the perspective of optimal technology enhanced music learning are discussed.

## 3. Review of Current Educational Technologies

In this section, a list of computer software and mobile apps, chosen among the most popular for number of downloads and the most innovative systems created for music pedagogy, is analyzed and described. The software selected in alphabetical order are (refer to Table A1 for URL references): Anytune Pro+, Amazing Slow Downer, EarMaster, Estill Voiceprint Plus, forScore, GNU Solfege, Guitar Pro, GuitarToolkit, GuitarTuna, KORG cortosia, Knock Box Metronome, Modacity: Pro Music Practice, liveBPM—Beat Detector, Piascore, QuantiForce, Rec'n'Share, Rhythm Teacher, Rhythm Trainer, Riyaz, RTFactory Rudiments, SkyNote, SmartMusic, Tempo, The Metronome by Soundbrenner, TonalEnergy, tonestro, Visual Note, Yousician. These edTech systems offer functionality normally applicable to all categories of musical instruments, with some exceptions for technologies dedicated to plucked strings (i.e., Guitar Pro, GuitarToolkit, GuitarTuna, Visual Note, Yousician), to percussion (i.e., liveBPM, Knock Box Metronome, RTFactory Rudiments) or winds and bowed strings (i.e., KORG cortosia, QuantiForce, tonestro). Some of the systems provide flexibility in terms of expected proficiency level, allowing the learning goals and exercise levels to be modified as the student progresses.

As mentioned in section 1, an inclusive list of software in support of music education is provided by Axford (2015) in a 250+ page book published in 2015 that is now partially outdated, given the high birth and death rate of these technologies. For this reason, we prefer to avoid the replication of a similar updated work, but to focus on the classification of the pedagogical aspects addressed. Thus, we have chosen to present a comprehensive list of software across the range of provided functionality and adopted hardware components. Within each category, we select the most popular—in terms of number of downloads—or innovative systems reported in publications.

### 3.1. Classification Based on Functionalities

Table 1 provides a list of the computer software and mobile apps considered in this study. The categories adopted for the classification are described below.

**Table 1:** List of software in support of music instrument learning classified according to the provided macro-functionalities.

**Software**	**Digital**	**Metronome**	**Tuner**	**Rhythmic**	**Sound**	**Fingering**	**Performance**	**Statistics**	**External**
	**score**			**refinement**		**display**	**feedback**		**hardware**
*Piascore*	✓	✓	✓						
*forScore*	✓	✓	✓						
*Modacity*		✓	✓					✓	
*GuitarToolkit*		✓	✓			✓			
*Guitar* *Pro*	✓	✓				✓	✓		
*GuitarTuna*		✓	✓			✓			
*Visual* *Note*		✓	✓			✓	✓		✓
*SmartMusic*	✓	✓	✓			✓	✓	✓	
*tonestro*	✓	✓	✓			✓	✓	✓	
*Yousician*	✓	✓	✓			✓	✓	✓	
*TonalEnergy*		✓	✓		✓				
*KORG* *cortosia*		✓	✓		✓				
*QuantiForce*					✓				✓
*Riyaz*−*Learn* *to* *Sing*		✓	✓		✓		✓	✓	
*Vocal* *Pitch* *Monitor*		✓	✓		✓				
*Estill* *Voiceprint* *Plus*			✓		✓				
*SkyNote*	✓	✓	✓		✓		✓		✓
*GNU* *Solfege*		✓		✓					
*EarMaster*	✓	✓	✓	✓			✓	✓	
*liveBPM*				✓			✓	✓	
*Tempo*		✓	✓	✓					
*Anytune* *Pro*+				✓					
*Amazing* *Slow* *Downer*				✓					
*Rec*′*n*′*Share*				✓					
*Rhythm* *Trainer*				✓					
*Knock* *Box* *Metronome*		✓		✓					
*RTFactory* *Rudiments*	✓			✓				✓	
*Soundbrenner*		✓		✓			✓		✓

#### 3.1.1. Digital Score Rendering

All software applications in this category provide a score in Western diastematic notation. The musician can add annotations as on a paper score (i.e., forScore, Piascore), play by turning the page through a specific functionality (e.g., foot switchers, touch pad, wink detection on camera), write in musical notation directly by playing the instrument (i.e., Guitar Pro), or following the score on a rolling window. Such software can also keep track of how much time the user spends on each exercise, allowing statistical calculations on the distribution of study time. While applications in this category do not directly assist with pedagogy, they provide a useful and popular functionality in music performance, especially as digital versions of music scores become prevalent. This type of software contains pedagogical potential especially when embedded in larger-scale systems that include algorithms to analyze the performer's sound in parallel and provide visualization or feedback on musical skills. A popular program in this category includes forScore, which offers the possibility to read PDF scores, organize music through metadata, build set lists, annotate, rewrite lyrics, add music notation, share, download and edit the scores, as well as providing metronome, tuner, and MIDI keyboard functionalities.

#### 3.1.2. Metronome and Basic Rhythm Functionalities

This category includes software systems that provide metronome functionality. This can be implemented according to its standard application by marking every beat, playing rhythmic structures of more complex subdivisions (i.e., Soundbrenner, TonalEnergy), detecting the metronomic tempo through tapping (i.e., KORG cortosia, Soundbrenner, TonalEnergy), illuminating the correct fingering in time (i.e., Visual Note), or verifying in real time the rhythmic accuracy of a musical performance on a given score (i.e., EarMaster, Riyaz, SkyNote, SmartMusic, tonestro, Yousician).

#### 3.1.3. Tuner Functionalities

Technologies included in this category provide tuner functionality. It can be implemented to facilitate the intonation of strings (i.e., GuitarToolkit, GuitarTuna, TonalEnergy, Visual Note, Yousician), as a chromatic tuner (i.e., EarMaster, forScore, GuitarToolkit, Modacity, Piascore, SmartMusic, TonalEnergy, Visual Note, Vocal Pitch Monitor), to tune on tuning systems other than equal temperament (i.e., Riyaz, TonalEnergy), to tune drums (i.e., Tempo), or to check the accuracy of the pitch of a musical performance on a given score (i.e., EarMaster, Riyaz, SkyNote, SmartMusic, tonestro, Yousician). For example, tonestro “listens” to a student playing along with a given (or purchased) score and provides feedback when pitches or rhythms are incorrectly executed.

#### 3.1.4. Systems That Assist With Advanced Rhythmic Refinement Skills

Software in this category offer exercises to improve rhythmic skills, such as rhythmic solfeggio tapping with the finger or clapping (i.e., EarMaster, GNU Solfege, Rhythm Trainer), identifying the metronomic value through sound analysis in real time (i.e., liveBPM) and offline (i.e., Rec'n'Share), setting tempo changes and rhythm patterns with increasing speed at any given number of beats (i.e., RTFactory Rudiments, Tempo), changing the tempo of an audio track (i.e., Amazing Slow Downer, Anytune Pro+, Rec'n'Share), setting cycles in which the metronome plays intermittently to check if the tempo is maintained during the absence of the beats (i.e., Knock Box Metronome), or providing rhythmic pulses on wearable hardware (i.e., Soundbrenner).

#### 3.1.5. Systems That Assist in the Technique and Control of Sound Production

This category includes features that provide an analysis or visualization of sound characteristics and technical aspects other than pitch, such as vibrato (i.e., Riyaz, Vocal Pitch Monitor), sound spectrum (i.e., Estill Voiceprint Plus, TonalEnergy), articulation and timbral characteristics (i.e., KORG cortosia, SkyNote), bow and brass mouthpiece pressure (i.e., QuantiForce), or posture and bow control (i.e., SkyNote). An interesting application in this category, KORG cortosia, was developed through a collaboration between KORG Inc. and Pompeu Fabra University (Bandiera et al., 2016). It provides an evaluation of what is defined as sound “goodness” by rating in real time five elements: pitch stability, dynamic stability, timbre stability, timbre richness, and attack clarity.

#### 3.1.6. Fingering Display

All software applications in this category provide correct fingering to play a specific note or chord. It can be displayed in the form of a chord library (i.e., GuitarToolkit, GuitarTuna), on a rolling score window in real time (i.e., Guitar Pro, Yousician), offline (i.e., SmartMusic, tonestro), or by illuminating the keys *via* a purchased external hardware component (i.e., Visual Note). A popular software in this category includes Yousician, which illustrates the appropriate fingering on a scrolling window in real time with the performance of a song. For plucked string instruments, it shows which string should be plucked, the corresponding fret number to press, and different colors recommend which finger to use for playing the note. In case there are different alternative fingerings for playing the same note or the same chord, Yousician suggests the most convenient solution to perform the specific song more easily.

#### 3.1.7. Systems Providing Feedback on Music Performances

This category includes functionalities that display, monitor and/or assess the correctness of a music performance. The implementation of these functionalities is coupled with algorithms that check the accuracy of rhythm and pitch (i.e., Guitar Pro, Riyaz, SmartMusic, Tonestro, Yousician), timbre and articulation (i.e., SkyNote) for a given score to provide an overall grade of the performance. This type of software is generally applied to the overall evaluation of pieces from the repertoire of performance and musical expression. However, alternative applications can be found dedicated to individual technical aspects, such as monitoring tempo (e.g., LiveBPM, Soundbrenner) and indicating fingering (e.g., Visual Note) in real time.

#### 3.1.8. Systems Applying Statistical Models to Keep Track of the User's Proficiency

Software in this category collect data on performances, displaying or analyzing them according to specific parameters, and store and process the results over time by applying statistical models to illustrate the progress of the musician (i.e., EarMaster, Riyaz, RTFactory Rudiments, SmartMusic, tonestro, Yousician). For example, EarMaster provides a window interface where users can visualize their achieved results and the time spent on each exercise, to help them monitor their progress and analyze strengths and weaknesses. The statistics functionality is also used to provide a visualization of a specific parameter over a short period of time for a single performance (i.e., liveBPM).

#### 3.1.9. Systems Requiring External Hardware

This category highlights technologies that rely on dedicated hardware components, instead of using the built-in sensors of PCs and smartphones. They can include cameras to provide indications about posture and bow tilting angles through motion capture techniques (i.e., SkyNote), wearable devices (i.e., the Soundbrenner metronome smartwatches), force transducers (i.e., QuantiForce), or LED lighting systems (i.e., the LED keyboard adapter for guitar proposed by Visual Note).

### 3.2. Classification Based on Hardware Components

In Table 1, a set of macro-functionalities for technology enhanced music learning is represented. An alternative classification consists in subdividing the aforementioned software according to the hardware components used:

- **Graphic display**: Many software systems use a graphic display to illustrate sheet music, show fingerings, provide light pulses as metronome indication, and generally explain the software functionalities. Some systems also use touch displays, for example, to add annotations or determine rhythmic information by finger tapping.
- **Microphone**: Systems that record audio signals for further processing and display make use of microphones in order to extract specific sound information, such as the fundamental frequency, onset detection, spectral descriptors for timbral information retrieval, articulation, vibrato, and loudness metering.
- **Speaker**: Some systems output audio signals through speakers, such as metronome ticks, edited audio tracks or melodic and harmonic accompaniment.
- **Camera**: Visual information can be collected using a camera in order to provide indications about posture and bow tilting angles through motion capture techniques or detect specific cues, such as winks, to turn page.
- **Other hardware components**: The software systems previously mentioned in the external hardware category all make use of non-standard hardware components not provided on PCs or mobile devices.

This further classification clearly indicates how the development of this type of software has tried to exploit the use of built-in sensors normally installed in PCs or mobile phones. Although software programming and calibration difficulties may be introduced, this choice is largely justified by the difference in marketing costs. Indeed, the cost of the software highlighted in the rightmost column of Table 1 exceeds by more than one order of magnitude the cost of software that rely on already installed built-in components.

Music pedagogy software systems that support audio and video recording of performances for subsequent analysis by students or teachers (e.g., Modacity) are not considered in Table 1. These systems allow students to externally identify weaknesses that need improvement and develop their own critical sense. Although this technology is still under-used, it offers very promising pedagogical potential for students of music (Fautley, 2013). However, this study intends to consider systems whose support and feedback are provided by the technology itself through the implementation of dedicated algorithms (and not as subjective judgments provided by the user).

Other categories of functionalities useful for music learning not included in Table 1 are the inclusion of videos on educational courses and masterclasses [e.g., Pickup Music^1^, Riyaz, tonestro, TrueFire (see text footnote 1), Youtube (see text footnote 1)] or the availability of a platform to receive individual private lessons *via* video with professional teachers [e.g., Play with a Pro (see text footnote 1), Riyaz, tonestro]. However, in this case the technology is used just as a communication platform to carry out live or recorded music lessons with a human teacher. This category is beyond the scope of this study, which intends to analyze an exclusive relationship with technology that the student can turn to and rely on during practice sessions in between visits to their instructors. Since music lessons for beginners typically take place once a week, we believe that the individual practice sessions between lessons contain a high learning potential which, when exploited effectively, can improve and speed up the overall learning experience.

Although the list of software examined is far from exhaustive, the described classifications give an idea of the state of the art on how software supporting music pedagogy are structured and what types of algorithms and technologies they implement. Section 4 discusses the classifications provided, identifies their possible limitations and proposes future directions of technologies for music instrument pedagogy.

## 4. Discussion

The software survey and classification demonstrates the extent to which the metronome and tuner have been widely adopted by nearly all current music pedagogy technologies. They are implemented in most of the systems considered in Table 1, indicating a high level of perceived usefulness. Initially implemented on dedicated hardware devices, the metronome and tuner functions were integrated into PC software or mobile apps, using their built-in components. Despite the huge technological advancement of the Digital Revolution, the functionalities of the metronome and tuner are clearly considered essential in music learning contexts.

We believe that the widespread use of metronome and tuner stems mainly from the fact that they are focused on teaching or assisting with an abstract technical concept. The metronome provides an audible indication of the tempo the player has to maintain during the performance, while the tuner provides a visualization of the fundamental frequency played, comparing it to a previously selected reference frequency. Such tools help the musician to understand musical concepts that are often difficult for performers to consistently internalize or perceive. By clearly understanding the technical concept and then the musical goal to be pursued through an audiovisual learning approach, students can therefore considerably improve the quality of their practice sessions and internalize more quickly a correct way of playing. Thus, music students develop and improve procedural memory, which allows them to learn movements, habits and skills almost independently of their conscious thought (Squire, 1992). These skills, learned automatically and internalized correctly, guarantee musicians a solid and effective technical background on which to rely during the performance and allow them to improve response and recovery to mistakes during performance (Lam, 2020). In fact, being based on abstract concepts, the metronome and tuner can be effectively applied in flexible ways and without particular limitations in most performance contexts, demonstrating their universality of application.

### 4.1. Current Limitations

The widespread use and perceived usefulness of the metronome and tuner in music pedagogy has inspired numerous other musical software, as previously surveyed, which have focused on developing their application on predetermined musical scores drawn from the repertoire of performance and musical expression. Indeed, current developments in many software systems have focused on expanding the metronome and tuner functionalities to provide real-time feedback on pitch and rhythm correctness during the performance of a given musical score (i.e., Guitar Pro, Riyaz, SkyNote, SmartMusic, Tonestro, Yousician). By applying an objective judgment on the accuracy of rhythm and pitch, these software offer an evaluation of the overall musical performance. However, the adoption of this technological method in the field of music education for beginners may present significant limitations to the effectiveness of their pedagogical experience:

- This type of music software, which evaluate the correct pitch and rhythm, can give the false impression that to play well and be a good musician it is sufficient to play the right notes and in time. However, this is obviously not true. A good musician is a performer capable of communicating emotions through sound, drawing on their wealth of technical skills developed and refined over time. While the musician needs to execute the notes and rhythms correctly, artistic expression fundamentally involves often subtle deviations from exact rhythmic or pitch accuracy. The attention of the performance should be mainly linked to the expressiveness and communication of emotions with the audience (which normally varies according to the type of audience, their response, the acoustics of the environment, the type of concert, etc.); the overall quality of the performance is therefore less suitable to be judged by the software, but rather by human sensitivity. In fact, musicians are granted a flexibility of expression within the technical rules to be less rigid and more communicative. This is one of the main differences between a mere MIDI performance and an artistic interpretation.
  Informal experiments with tonestro, for example, have shown that a very inexpressive performance, in which the notated dynamic and articulation marks were ignored, can achieve very high scores. On the other hand, more expressive musical performances with proper attention to notated articulations and dynamics generally earn poorer scores.
- If the software provides an evaluation of the performance by rigidly judging rhythmic and pitch correctness on a note-by-note basis, according to a subtractive method of judgement (i.e., each error lowers the overall judgement score), the musician's attention will be focused on playing correctly each note in order to achieve the highest final score. This can inhibit the expressiveness of the performer, who concentrates on playing note by note in a pedantic manner, breaking up the melody, instead of artistically playing longer and more expressive musical phrases.
  Moreover, all this can cause an incorrect approach to performance, especially for beginners, who have not yet developed a solid personal style of expression. Musicians become more focused on receiving positive feedback from the software, trying to avoid the appearance of red error marks in the display, rather than trying to express their musical ideas by seeking empathetic contact with the audience. This approach to performance, based on trying to avoid mistakes instead of proposing musical ideas and communicating emotions, can even generate tensions in musicians that ultimately affect their wellbeing.
- Some of the reviewed software follows student progress through statistical analysis of their score. Implementing statistical models applied to collected data to generate a learning curve over time is an effective way to identify strengths and weaknesses for targeted practice. However, this indication is not pedagogically relevant if the software expects the musician to sound like a robot.

Despite these potential limitations in the pedagogical experience for beginners, such software offers powerful playful and entertaining aspects for music players, which greatly encourages user motivation. In particular, the aspect of playing along with backing tracks leads the musician to imagine playing together with others, bringing a deeper involvement in the experience, although the feedback component still continues to present the aforementioned drawbacks.

Another barrier to the adoption of technology within music courses might be represented by ineffective and overly-complicated interfaces. For example, KORG cortosia is one of the few software systems that intends to address different technical aspects beyond rhythm and pitch: pitch stability, dynamic stability, timbre stability, timbre richness, and attack clarity. Although the idea of tackling different technical skills within a single app is compelling, it is severely limited in terms of the interface. The KORG cortosia software shows a five-axis view, each associated with the five different skills considered, and provides an overall numerical score averaged over those five parameters. It is therefore complicated to isolate one parameter at a time, and it is difficult for a student to focus on and manage five at once. For example, a student may need to study pitch stability while playing a crescendo or diminuendo, without the overall numerical score being affected due to changes in dynamics.

Furthermore, even if the functionality of isolating one parameter at a time were easily accessible, a numerical score may not be the most pedagogically effective way to provide feedback. For example, wind instrumentalists need to develop different types of attack or articulation, using different pronunciations, to fulfill equally varied musical needs. It is therefore difficult to implement an algorithm that gives a consistent judgment of attack clarity for all types of attacks. A generic numerical score on this technical skill may not give the students a clear understanding of what they are doing wrong and how to fix the problem. This type of feedback easily risks confusing the students further. A visualization of sound initiation, on the other hand, is much more effective from a teaching point of view, because it allows musicians to associate an image with the execution of a technical skill, and once they understand how the interaction between their body and the musical instrument affects the image, the student has the opportunity to understand how to self-correct and improve. Moreover, a visualization provides flexible feedback that can be adapted to give useful information about different types of a technical skill. For example, a wind musician may associate different images with different types of attack and, by seeking out those images during practice sessions, gain greater clarity on how to manage and master the various articulations.

Other examples of software with possible interface problems are the timbral indications of Estill Voiceprint Plus and TonalEnergy. These systems illustrate the evolution of the audio spectrum over time or the height of harmonic peaks in real time in order to provide indications of the timbral quality of the sound. The sound spectrum and its relative harmonic distribution contain important information about the correctness of the sound produced. An unnatural or strained sound may indicate the presence of muscular rigidity in a wind performer and inefficiency in playing (Thompson, 2003; Jacobs and Nelson, 2006). However, being able to extract this information by referring only to the spectrogram and its harmonic distribution is a difficult or almost impossible task for a music student.

These difficulties in analyzing particular technical abilities—such as timbre quality or technical skills considered by Estill Voiceprint Plus, TonalEnergy, and KORG cortosia—are further accentuated by the fact that these software systems analyze audio data collected by microphones embedded in PCs or mobile devices. The recorded audio signal therefore depends on the particular model of microphone sensor installed (usually not suitable for recording musical instruments with sufficient quality), on the distance and position of the microphone with respect to the sound source, and on the acoustics of the room. For example, if a trombone player changes orientation or places the smartphone behind the bell in order to better see the display, the feedback provided by the software will be altered compared to when holding the smartphone in front of a stationary bell, making the system unrepeatable and unreliable. In fact, sound dynamics is a determining factor in identifying the timbral properties of an instrument (Fabiani and Friberg, 2011).

Another limit to the creation and production of technologies for music instrument learning involves the cost and complexity of necessary external hardware components. SkyNote, for example, presents excellent goals regarding what we believe can support music pedagogy. However, the project never left the research phase to find a real application in music classrooms, as it requires hardware equipment that is too expensive and sophisticated to be easily obtained and installed by a music student.

In the next subsection, we propose possible directions for technologies in support of music pedagogy that address the limitations mentioned above.

### 4.2. Future Directions

Given the issues discussed in the previous section, a sensible direction for the development of new pedagogic software systems is to focus on teaching a specific technical concept in an “exercise-like” context (in comparison to a context in which the player may be inclined to be musically expressive). In this way, the musician learns the technical skill in a universal context and, once internalized, can apply it confidently to any performance without incurring the aforementioned risks and limitations. Considering feedback and visualization on a technical aspect, rather than a performance, allows the system to provide higher accuracy and reliability, given fewer variables involved in software development. By focusing on a specific technical skill, players are expected to play like a robot, in order to train their muscle memory through deliberate practice. Systems designed in this way would have a type of functionality that is similar to the metronome and tuner.

There are other technical aspects besides pitch and rhythm that can be addressed with newer technologies and a development in this direction could open new ways to improve musical pedagogy. These technical abilities are generally more dependent on the particular musical instrument played, requiring greater specificity of the parameters analyzed and provided by the system. Here lies significant potential that is still under-explored in the field of technology-enhanced music learning.

Skills which are fundamental for the optimal technical control of a musical instrument include for example dynamics, vibrato, articulation, staccato/tonguing, sound resonance, body setting (e.g., efficient embouchure, bow, and sticks handling), or legato quality. Some of the software listed in the Table 1 pursue this direction, although in some cases their pedagogical potential may face the mentioned limitations. In the following, a selection of addressed technical aspects, are analyzed and discussed.

#### 4.2.1. Vibrato

Some systems provide a visualization of the evolution of the fundamental frequency or sound spectrum over time (e.g., Vocal Pitch Monitor, Estill Voiceprint Plus). This provides visual indications of the amplitude, frequency and extent of the vibrato.

Possible applicable extensions to these features could include interactive exercises that assess control of these parameters. For example, a system could specify a sequence of long notes, embedded in a rhythmic context, that the performer has to play at predetermined vibrato patterns (e.g., duines, triplets, quatrains at each beat) within specific frequency and amplitude threshold values. Training on these exercises would allow the musician to learn vibrato control under different conditions and master this skill from a technical standpoint. In this way, when performers later want to expressively interpret a piece of music (e.g., aria, sonata, cantata), they will have the flexibility to produce the type of vibrato they feel is most appropriate for that performance, without being constrained by technical limitations.

#### 4.2.2. Attack Clarity

Attack clarity refers to the purity or accuracy of the onset of a sound, especially with respect to achieving the desired fundamental frequency that is not contaminated by noise or undesired frequency components. Attack clarity may involve different characteristics depending on the musical instrument considered. Optimal articulation usually requires a very short time duration between the silence before the attack and the achievement of a fully developed sound, regardless of the particular type of articulation, dynamics, or accent required.

Among the software systems listed, some of them (i.e., TonalEnergy, Vocal Pitch Monitor, Estill Voiceprint Plus) include useful features to provide a visualization of this skill. They in fact provide a display of the evolution of the fundamental frequency or spectrum over time. By looking at the graphs, musicians can partially verify the accuracy of their articulation. However, this functionality could be greatly improved by providing detailed visualization of the attack of the notes produced, using short time windows to analyze the audio signal. Also, it could be very useful to provide feedback (e.g., in milliseconds) on the time duration used to achieve a relatively stationary sound from a timbral point of view.

Advanced sound processing algorithms implemented in SkyNote, within the TELMI project^2^, have been developed to identify different types of violin pronunciation (e.g., staccato, martelé, détaché) (Ramirez et al., 2018; Giraldo et al., 2019). However, the project has not yet found use in music pedagogy, as it has remained in the research phase.

#### 4.2.3. Dynamics and Timbre Characteristics

Some software provide a real-time display of the sound spectrum or of the harmonic energy content, through which the musician can search for specific timbral characteristics and dynamics (e.g., TonalEnergy, Estill Voiceprint Plus). However, as discussed in section 4.1, their application in music pedagogy is limited due to feedback interpretation difficulties and because the audio recording conditions of a mobile device microphone in a practice room is not guaranteed to provide sufficient levels of repeatability and accuracy. An attempt at interpretation is provided by SkyNote, but only still in an exploratory research setting.

To provide feedback based on timbral characteristics, we might suggest to use a dedicated external microphone that has a configuration to be installed at the same distance and position from the sound source (e.g., clip-on microphones). In this way, the variable of dynamics, which is crucial for the identification of the timbral properties of a sound (Fabiani and Friberg, 2011), is normalized. With this solution, more robust software algorithms can be developed that rely on the recording characteristics of a single microphone sensor, suitable for recording musical instruments, instead of relying on recordings taken from several microphones, usually optimized for voice calls, embedded in different devices. In this case, the additional cost of having to use an external hardware component is justified by the improved reliability of the overall system. The adoption of such a microphone would open the opportunity to provide feedback and visualizations on the sound dynamics produced and on timbral aspects for which a higher quality recording is required.

#### 4.2.4. Relative Tuning

Standard tuners represent useful tools to develop a consistent intonation through intervals, scales, dynamics, and articulation. However, players of variable-intonation pitched instruments (e.g., violin, trombone) must adjust their pitch relative to that of others when performing in ensemble music contexts. It is therefore important that students of these musical instruments develop the ability to listen to the sound of others as they play, understand how much it differs from their own pitch, and correct any discrepancies.

We believe that modern technology has the potential to help musicians of these instruments develop this skill, using graphic displays, microphones, and headphones. It would help future students better integrate into ensemble music groups, more easily find a common pitch, and generally better control the dynamic balance of their sound.

In summary, this study intends to highlight the scarcity of low-cost technologies that provide visualization and feedback on the technical concepts necessary for a complete learning of a musical instrument, as the metronome and the tuner do. Their development, coupled with data collection and statistical analysis capabilities to monitor the level of the musician in their respective technical skills, would provide significant support to visualize progress over time, identify effective practice routines, and method of study, as well as represent important tools for stress management and improving performance wellbeing.

## 5. Conclusions

This study presents a review of the main features provided by the technological tools that have been developed to support music instrument learning and investigates their potential benefits and utility. The widespread success of the metronome and tuner have prompted the subsequent development of numerous software and mobile applications that attempt to go beyond basic rhythm and pitch accuracy. However, their use in applied performance repertoire contexts, where the system makes an evaluation that discourages artistic expression, can present important drawbacks in pedagogical experience especially for beginners, who generally have less technical control and sense of self-evaluation.

There are numerous other facets of learning to master an instrument that are still poorly addressed by current music technologies, such as control of dynamics, attack and release precision and refinement, flexibility with timbre, vibrato, embouchure configuration and variation, finger position and movement, posture, and breathing, to name a few. We believe that the development of new technologies that provide visualization or perception of technical concepts related to the learning of a specific musical instrument may find broad use in music practice rooms, if they are relatively cheap and have user-friendly interfaces. Clearly understanding a musical concept to be researched and pursued in individual study sessions through audiovisual systems can consistently help instrumentalists in becoming more efficient with their practice. In addition, such systems would represent objective yardsticks for teachers to verify proposed recommendations and improve lesson effectiveness.

By suggesting these new directions for future assistive technology supporting music pedagogy, we hope to better connect the field of technology development with the music school community so that students can enjoy a more fulfilling artistic experience.

## Author Contributions

AA: conceptualization, survey, discussion, visualization, writing—original draft, and review and editing. GS: supervision, discussion, visualization, and review and editing. Both authors contributed to the article and approved the submitted version.

## Funding

Partial funding for the present study has been provided to AA by a Tomlinson Doctoral Fellowship from McGill University. Further funding has been provided by a Discovery Grant from the Natural Sciences and Engineering Research Council of Canada (RGPIN-2020-04874).

## Conflict of Interest

The authors declare that the research was conducted in the absence of any commercial or financial relationships that could be construed as a potential conflict of interest.

## Publisher's Note

All claims expressed in this article are solely those of the authors and do not necessarily represent those of their affiliated organizations, or those of the publisher, the editors and the reviewers. Any product that may be evaluated in this article, or claim that may be made by its manufacturer, is not guaranteed or endorsed by the publisher.

## Footnotes

^1^Refer to Table A1 for URL references.

^2^ http://telmi.upf.edu/

## Appendix

**Table A1:** List of considered software with corresponding URL (accessed December 11, 2021).

**Software**	**Reference link**
Anytune Pro+	https://anytune.us/
Amazing Slow Downer	http://www.ronimusic.com/asdiphone.htm
EarMaster	https://www.earmaster.com/
Estill Voiceprint Plus	https://store.estillvoice.com/collections/clinical-software/products/voiceprint-plus-cd-mac-edition
forScore	https://forscore.co/
GNU Solfege	https://www.gnu.org/software/solfege/solfege.html
Guitar Pro	https://www.guitar-pro.com/c/15-guitar-pro-ios-android
GuitarToolkit	https://apps.apple.com/ca/app/guitartoolkit-tuner-metronome-chords-scales/id284962368
GuitarTuna	https://yousician.com/guitartuna
KORG cortosia	https://www.korg.com/us/products/software/cortosia/
Knock Box Metronome	https://www.pfeiferdrumco.com/knock-box-metronome.html
Modacity	https://www.modacity.co/
liveBPM - Beat Detector	https://play.google.com/store/apps/details?id=com.DanielBach.liveBPM
Piascore	http://piascore.com/
Pickup Music	https://www.pickupmusic.com/
Play with a Pro	https://www.playwithapro.com/
QuantiForce	https://www.bonsai-systems.com/music-tech/
Rec–n–Share	https://ca.yamaha.com/en/products/musicalinstruments/drums/eldrums/apps/recnshare/index.html
Rhythm Teacher	https://play.google.com/store/apps/details?id=net.gamya.rhythm
Rhythm Trainer	https://play.google.com/store/apps/details?id=ru.demax.rhythmerr
Riyaz	https://riyazapp.com/
RTFactory Rudiments	https://rhythmtoolsfactory.de/appsios/rudimentsios/
SkyNote	http://telmi.upf.edu/
SmartMusic	https://www.smartmusic.com/
Tempo	http://www.frozenape.com/tempo-metronome.html
Soundbrenner	https://www.soundbrenner.com/
TonalEnergy	https://www.tonalenergy.com/
tonestro	https://www.tonestro.com/
TrueFire	https://truefire.com/
Visual Note	https://www.visual-note.com/
Yousician	https://yousician.com/
Youtube	https://www.youtube.com/
